# Self-Perceived Physical Level and Fitness Performance in Children and Adolescents with Inflammatory Bowel Disease

**DOI:** 10.3390/children9091399

**Published:** 2022-09-15

**Authors:** Francesca Penagini, Valeria Calcaterra, Dario Dilillo, Matteo Vandoni, Laura Gianolio, Alessandro Gatti, Giulia Rendo, Matteo Giuriato, Lucia Cococcioni, Annalisa De Silvestri, Gianvincenzo Zuccotti

**Affiliations:** 1 Pediatric Department, “Vittore Buzzi” Children’s Hospital, 20154 Milan, Italy; 2 Department of Internal Medicine, University of Pavia, 27100 Pavia, Italy; 3 Laboratory of Adapted Motor Activity (LAMA), Department of Public health, Experimental Medicine and Forensic Science, University of Pavia, 27100 Pavia, Italy; 4 Department of Neurosciences, Biomedicine and Movement Sciences, University of Verona, 37100 Verona, Italy; 5 Faculty of Physical Culture, Unit of Molecular Biology, Department of Health and Natural Sciences, Gdansk University of Physical Education and Sport, 80070 Gdansk, Poland; 6 Biometry & Clinical Epidemiology, Scientific Direction, Fondazione IRCCS Policlinico San Matteo, 27100 Pavia, Italy; 7 Department of Biomedical and Clinical Science, University of Milano, 20157 Milano, Italy

**Keywords:** inflammatory bowel diseases, exercise, fitness performance, children, physical activity, cardiovascular risk, metabolic

## Abstract

Background: Inflammatory bowel disease (IBD) patients show a higher risk of developing metabolic and cardiovascular diseases due to the presence of systemic low-grade chronic inflammation. Exercise can improve cardiovascular fitness and modulate the inflammatory processes. We evaluated the physical activity (PA) level and the fitness performance of children and adolescents with IBD. Patients and methods: We considered 54 pediatric patients with IBD (14.6 ± 2.2; 22 M), including CD (n = 27) UC (n = 24) and IBD unclassified (n = 3), and 70 healthy children. In all children, the Physical Activity Questionnaire (PAQ-C) and the International Fitness Enjoyment Scale were self-reported and recorded. Results: PAQ-C showed significant difference in PA levels in patients with IBD compared to controls (*p* < 0.001). A decrease in general fitness (*p* = 0.003), cardiorespiratory fitness (*p* = 0.002), strength (*p* = 0.01), speed agility (*p* = 0.003), and flexibility (*p* = 0.01) were also detected between patients and controls. Speed agility was related to age (*p* = 0.02) and BMI z-score (*p* = 0.01), and flexibility to BMI z-score (*p* = 0.05). We noted a correlation between PA levels and physician global assessment (*p* = 0.021) and activity disease severity (*p* = 0.025). Conclusions: A poorer PA level and poor physical competence were found in patients with IBD compared to healthy children and adolescents. Monitored exercise could provide multiple benefits at both physical and psychological levels.

## 1. Introduction

Inflammatory bowel disease (IBD) represents a group of chronic inflammatory disorders that affect the gastrointestinal tract and includes Crohn’s disease (CD) and ulcerative colitis (UC) [1,2]. Approximately 25% of IBD patients present before the age of 20; among children with IBD, 4% present before 5 years of age and 18% before 10 years of age, with the peak onset in adolescence [1].

Even though the etiology of the disease remains not fully elucidated, literature data proposed that genetic susceptibility, environment, gut microbiota alteration and immunological factors are involved in the pathogenesis [1,2]. The clinical course of IBD is heterogeneous, with some patients following a mild course while others experience an aggressive progression of disease [1,2].

Metabolic disorders and cardiovascular disease (CVD) may affect the disease course and prognosis in adults with IBD, increasing the risk of early morbidity and mortality [3]. The pathogenic association between these complications has not been fully elucidated; however, a link between cardiometabolic risk and chronic inflammation has been proposed in affected patients [4,5].

Chronic disorders such as type 2 diabetes, CVD, and IBD share common features in their pathology; all of them are characterized by systemic low-grade chronic inflammation (SLGCI) [4].

In IBD, the disruption of the intestinal epithelium integrity appears to be an early phenomenon in the disease pathogenesis, allowing bacteria and their products to penetrate through a barrier leak and leading to an abnormal immunological and inflammatory response [6]. The induction of SLGCI is closely associated with human aging and, subsequently, with metabolic and cardiovascular derangement.

In fact, inflammation represents a beneficial process as an acute and transient immune response to harmful conditions; however, when it is chronic and of low grade, it leads to tissue dysfunction and degeneration [6,7,8,9]. Additionally, in IBD, a link between host metabolic disorders and gut microbiota dysfunction has also been reported [4,6].

Physical exercise (PE) is considered to be a non-pharmacological intervention that can improve cardiovascular fitness and modulate inflammatory processes, reducing health risks starting from childhood [9,10]. The physical activity (PA) guidelines of the World Health Organization (WHO) recommend that children and adolescents aged from 5 to 17 years old engage in at least 60 min of moderate to vigorous play or sport activities per day. Unfortunately, less than 20% of adolescents in the world are sufficiently physically active [10]. In Italy, only a limited part of the pediatric population complies with the PA guidelines set by the WHO, indicating a higher risk of developing cardiometabolic disorders. Previous studies have shown that PA levels are related to physical competence and self-perceptions of physical fitness. Children with poor physical competence and poor self-esteem tend to have a lower PA level compared to peers with a higher physical competence and higher self-esteem [11].

Data on the PA level of pediatric patients with IBD are limited [12,13,14,15]. PA levels and PA participation are lower in IBD pediatric patients compared to healthy children [12,13,14,15]. The most commonly reported barriers to PA participation are the intestinal or extraintestinal symptoms, the PA perception, and the psychological factors, such as depression [12,13,14,15]. Therefore, it is crucial to investigate the PA and perceived fitness level of this population to more effectively promote programs aimed at improving and ameliorating lifestyle and PA practices [16].

For this reason, we evaluated the self-perceived physical level and fitness performance of children and adolescents with IBD, compared to healthy children. PA planning could represent a crucial factor in the treatment of IBD and in the prevention of related cardiometabolic complications.

## 2. Patients and Methods

### 2.1. Patients

We consecutively enrolled 54 pediatric patients with IBD (aged 14.6 ± 2.2; 22 M), including CD (n = 27), UC (n = 24), and IBD-unclassified (IBD-U) (n = 3). The children and adolescents were referred to the IBD outpatient clinic at Vittore Buzzi Children’s Hospital of Milan, Italy, between 1 April and 15 June 2022. The inclusion criteria were an age between 8 and 16 years old and a comprehension of the Italian language. The exclusion criteria were a known non-comprehension of the Italian language and absolute contraindications to PA practice.

As controls, we considered 70 healthy children and adolescents comparable for age and sex (13.5 ± 2.2; 22 M) and referred for auxological evaluation during the enrollment period.

In all children, auxological evaluation and disease activity and severity was recorded. The Physical Activity Questionnaire (PAQ-C) and the International Fitness Enjoyment Scale (IFIS) were also self-reported and recorded.

The study protocol was conducted according to the guidelines of the Declaration of Helsinki and approved by the Institutional Ethical Committee of the hospital (protocol number 0034655; date of approval: 11 August 2020). After being informed about the nature of the study, the parents or guardians provided written informed consent to participate in the study.

### 2.2. Methods

#### 2.2.1. Anthropometric Features

The height, weight, BMI, and BMI z-score of all patients were measured. The weight was quantified with participants not wearing shoes and in light clothing, standing upright in the center of the scale platform (Seca, Hamburg, Germany) [17,18]. A Harpenden stadiometer, with a fixed vertical backboard and an adjustable head piece, was used to measure the standing height (Holtain Ltd., Cross-well, UK); during the measurement, the children and adolescents were in an upright position, without shoes, hands at sides, aligning the head in the Frankfort horizontal plane [17,18]. The BMI was calculated as body weight (kilograms) divided by height (meters squared) and was transformed into BMI z-scores using the WHO reference values [19].

#### 2.2.2. Inflammatory Bowel Disease Definition and Disease Activity Scores

##### Disease Definition

We distinguished three subtypes of IBD on the basis of clinical, endoscopic, and histological features: CD, UC, and IBD-U.

CD was diagnosed in the case of transmural, discontinuous, and often granulomatous inflammation affecting any part of the gastrointestinal (GI) tract from the mouth to the anus. UC was diagnosed in the case of continuous superficial mucosal ulceration limited to the colon and extending proximally from the rectum. IBD-U was diagnosed in the case of inconclusive clinical and endoscopic features with characteristics of either CD or UC.

##### Disease Activity Scores

Disease Activity Scores were used to assess the IBD severity. For CD, the Pediatric Crohn’s Disease Activity Index (PCDAI) was used [20]. The PCDAI comprises 11 items: symptoms (abdominal pain, stool pattern, and general wellbeing); physical examinations (the presence of perianal disease or extraintestinal manifestations); growth (weight and height); and serum inflammatory markers (hematocrit, erythrocyte sedimentation rate, and serum albumin). The PCDAI score ranges from 0 to 100 with higher scores indicating worse disease activity: <10 is consistent with inactive disease; 10–30 is consistent with mild disease; 31–40 is consistent with moderate disease; and >40 is consistent with severe disease [21,22]. For UC, the Pediatric Ulcerative Colitis Activity Index (PUCAI) was used [23,24]. The PUCAI is composed of six clinical items: abdominal pain; rectal bleeding; stool consistency; number of stools per day; nocturnal stools; and activity level. The PUCAI ranges from 0 to 85: <10 denotes remission; 10–34 indicates mild disease, 35–64 indicates moderate disease; and >65 indicates severe disease. Finally, for UC, CD, and IBD-U, the Physician Global Assessment (PGA) was used [25]. The PGA evaluates symptoms (e.g., abdominal pain, fatigue, diarrhea, and bloody stools), clinical signs (e.g., fistulas, weight loss, and abdominal tenderness), and laboratory tests. According to the PGA, IBDs can be classified as inactive, mild, moderate, and severe.

#### 2.2.3. The Physical Activity Questionnaire for Older Children (PAQ-C)

The PAQ-C is a self-reported questionnaire that evaluates the weekly amount of PA during the school year. This questionnaire was previously validated for school-aged children (approximately aged 8–14) and is appropriate for children currently in the school system. The PAQ-C has been proven as a valid and reliable measure of general PA levels from childhood to adolescence. The PAQ-C evaluates several domains of the daily habits of children such as the time spent on PA practice at school and PA performed in the afternoon or evening and in free time. The PAQ-C is cost- and time-efficient, easy to administer to large-scale populations, and displays normal distribution properties. The PAQ-C has been shown to have good reliability with an intra-class correlation (ICC) = 0.96 [26].

#### 2.2.4. The International Fitness Enjoyment Scale (IFIS)

The IFIS is a self-reported, simple, and short fitness scale. It has been previously validated in nine European countries and languages and defines physical fitness (PF) as an indicator of physical competence [27]. The IFIS consists of a 5-point Likert scale (from 1 (very poor) to 5 (very good) and contains questions focused on 5 macro-areas of fitness: general fitness; cardiorespiratory fitness; strength; speed agility; and flexibility. The IFIS has a high validity and moderate to good reliability (average weighted Kappa: 0.70 and 0.59, respectively) for school-aged children.

## 3. Statistical Analysis

All quantitative data were summarized as the mean and standard deviation (SD) or the median and IQR (range) as appropriate. The Shapiro–Wilk test was used to test the normality of the data. To test the differences between the groups and physical activity, a Student’s *t*-test or a one-way ANOVA was used. To take into account possible confounders, multivariable regression models were fitted using sex, age, and the BMI z-score as independent variables. The significance was set at a *p*-value less than 0.05. The enrolled sample achieves 80% power to identify as significant a difference between group means of about half standard deviation. All statistical analyses were performed using Stata software, version 17.0 (StataCorp, College Station, TX, USA).

## 4. Results

### 4.1. IBD Patients and Controls

The clinical features, PA level, and physical competence of the patients and the controls are reported in Table 1. All variables (except BMI z-score) are normally distributed. Age and sex distribution are not significantly different in patients and healthy children (*p* > 0.05). The BMI and BMI z-score were higher in the IBD patients compared with the controls (*p* < 0.001).

**Figure 1:**
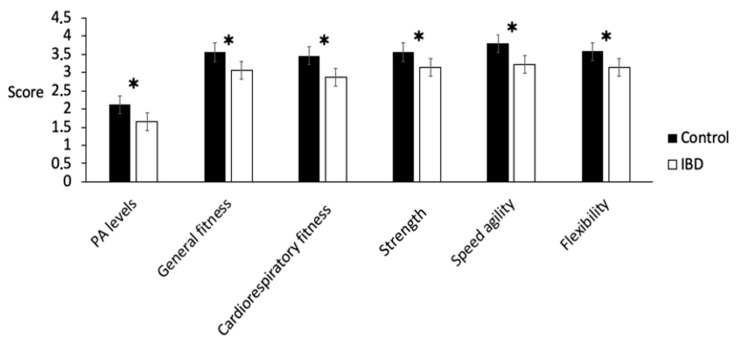
Physical activity levels and physical competences in patients affected by IBD (white columns) and in controls (black columns). Note: *p < 0.05.

As reported in Table 1 and Figure 1, the PAQ-C showed significant differences in the PA levels of patients with IBD compared with the controls (1.50 ± 0.05 vs. 2.06 ± 0.06; *p* < 0.001). The difference remained statistically significant after adjusting for sex, age, and BMI z-score.

PA levels were not correlated to age, sex, and auxological parameters (all *p* > 0.05).

A significant decrease in general fitness (3.05 ± 0.13 vs. 3.55 ± 0.10; *p* = 0.003), cardiorespiratory fitness (2.87 ± 0.14 vs. 3.45 ± 0.11; *p* = 0.002), strength (3.14 ± 0.14 vs. 3.55 ± 0.10; *p* = 0.01), speed agility (3.20 ± 0.16 vs. 3.78 ± 0.11; *p* = 0.003), and flexibility (3.12 ± 0.13 vs. 3.57 ± 0.12; *p* = 0.01) were detected between the children affected by IBD and the controls (Figure 1).

The difference remained statistically significant after adjusting for sex, age, and BMI z-score for cardiorespiratory fitness and strength.

Speed agility was significantly related to age (*p* = 0.02; beta coefficient = −0.11; [CI 95% −0.20; −0.01]), BMI z-score (*p* = 0.01; beta coefficient = −0.20; [CI 95% −0.36; −0.04]), and flexibility with the BMI z-score (*p* = 0.05; beta coefficient = −0.14; [CI 95% −0.30; −0.003]); no other relationships between physical competence and the clinical features were detected in the multivariable models.

### 4.2. Group of IBD and Severity Disease

A female and male predominance was noted in CD and UC, respectively (*p* < 0.001). The three groups—CD, UC, and IBD-U—were comparable for age (*p* > 0.05).

PUCAI/PCDAI scores showed inactive disease/remission in 42 patients (77.8%) and mild/moderate disease in 12 cases (22.2%), without any difference between sex and age (*p* > 0.05).

According to the PGA, IBDs patients were classified as inactive in 33 patients (61.1%), mild in 13 (24.1%), and moderate in 8 (14.8%) children, with a similar distribution between sex and age (all *p* = 0.95).

In Table 2, PA levels and physical competences in patients affected by IBD according to PGA and PUCAI/PCDAI were reported.

No significant differences in the PA levels (*p* = 0.59), general fitness (*p* = 0.19), cardiorespiratory fitness (*p* = 0.29), strength (*p* = 0.19), speed agility (*p* = 0.16), and flexibility (*p* = 0.44) were recorded between the patients affected by the different subtypes of IBD.

Independent from the subtype of IBD, we noted a correlation between the PA levels and the PGA (*p* = 0.021; beta coefficient = −0.28; [CI 95% −0.53; −0.04]) and the PUCAI/PCDAI Scores (*p* = 0.025; beta coefficient = −0.24; [CI 95% −0.45; −0.04]); these were also inversely related to age (*p* < 0.01; beta coefficient = −0.06; [CI 95% −0.10; −0.02]).

No relationship was observed between physical competence (including general and cardiorespiratory fitness, strength, speed agility, and flexibility) and the level of disease severity (all *p* > 0.05).

## 5. Discussion

PA is essential in the overall health status of children and adolescents [10]. The pediatric population benefits from the effects of PA with respect to PF, bone strength, cognitive functions, and psycho-social factors such as self-efficacy, self-worth, and self-esteem [28], also reducing anxiety and depression.

The clinical presentation in IBD pediatrics can be variable [1,2]. General symptoms, such as weight loss, fever, growth retardation, anorexia, or symptoms related to gastrointestinal tract including abdominal pain, constipation, diarrhea, rectal bleeding, and vomiting/nausea, can occur; additionally, extra-intestinal manifestations can be present [1,2]. IBD is a debilitating disorder characterized by cycles of disease activity and quiescence; the disease flares are unpredictable and occur in a random way [1,2,3]. The illness may limit PA in children and adolescents due to intestinal or extraintestinal manifestations, fatigue, or PA perception [29,30,31,32]; in particular, tiredness/fatigue and abdominal pain are described as the principal barriers to PA participation in both children and adults [31,32,33,34,35,36].

Although an impaired exercise capacity has been reported in adults with IBD, knowledge of the extent of this problem in pediatrics is limited [13,14,15].

Marchionni Beery et al. [29] showed that PA and sports participation in patients and parents was greatest before rather than after IBD diagnosis [30]. Bourdier et al. reported that levels of PA were lower in children with IBD than in their healthy counterparts [13]. Ploeger et al. demonstrated that both anaerobic and aerobic capacities were significantly lower in pediatric patients with IBD compared to the reference values from healthy children [14]. Godin et al. measured a lower PA in IBD patients compared to controls, with a reduced number of steps per day, a higher sedentary lifestyle, and a lower amount of time spent in moderate and vigorous activities [31].

We confirmed a poor level of PA and physical competence, such as general fitness, cardiorespiratory fitness, strength, speed agility, and flexibility, in IBD patients when compared with controls, without any differences between the different subtypes of disease. As reported by previous studies, a poor physical competence could reduce PA practice; for this reason, an enhanced evaluation of physical performance prior to engagement in sports activities could help to increase PA adherence and practice through the years.

In our population, PA levels were correlated with the PGA and severity of disease, supporting the theory that disease activity is a crucial deterrent for PA in these patients [13]. However, we also noted that the fitness performance of the affected children was not related to the level of disease severity, suggesting a plausible role of psychological factors as barriers to PA practice [30,32].

As reported, PA can contribute to an improvement in IBD symptoms and disease activity [30,33]. Although effective medical treatments exist for these chronic conditions (such as immunosuppressants and biologic agents), certain patients do not respond to medical treatments. Modifiable factors such regular PA may be considered as an adjunctive therapy regime to ameliorate the clinical response. To explore the pathogenic mechanisms by which PA impacts the clinical course of disease could be useful to offer a new preventive perspective in children and adolescents with IBD.

Disease-associated malnutrition, such as under- and overnutrition, is very common in children with IBD [33,34]. The etiology of perturbation of the nutritional status is multifactorial, including inflammatory response, drugs’ interaction, dysbiosis, alterations of energy expenditure and medical therapeutic interventions [34,35].

The double malnutrition burden may impact physical performance [36,37]. In our cohort of patients, we recorded that the BMI and BMI z-score were higher in the IBD patients than in the control group, without underweight patients (BMI z-score ≤ 2 SD); only four IBD patients had been recently treated with steroids. We noted that physical competence such as speed agility and flexibility were related to the BMI z-score. In our previous research, we observed that OB children showed lower PF (except for the upper body and explosive power); the speed performances were also lower in children with obesity [16]. The results were similar for both genders. Positive relationships were found between the ponderal indexes (body weight, fat mass, and BMI) and explosive performances. In contrast, children with a favorable body shape (low BMI, body weight, and stature) succeeded in the agility performances [1].

These findings may underscore the determinant role exerted by body mass and suggest the importance of monitoring body weight to preserve PF. Obesity represents a severe public health threat to children and adolescents [8,9]. In addition to the general risks associated with obese condition, obesity may have disease-specific risks in IBD children, influencing illness activity, long-term IBD morbidity and mortality, and medication interactions. Multidisciplinary resources, screening, counseling, and therapy should be the standard of care for the IBD patients to prevent obesity and related complications.

In the context of IBD, several beneficial effects of PE could be considered. Firstly, PA impacts on the cardiometabolic profile, influencing insulin sensitivity in adipose tissue, skeletal muscle, and the endothelium. PA also influences body weight and blood pressure, inducing changes in the HDL and VLDL cholesterol particle size [29]. Secondly, PA increases cardiac and skeletal muscle strength as well as inducing an improvement in maximal oxygen consumption, leading to an increased PA tolerance [8,9]. PA may also improve and attenuate systemic inflammation by the release of cytokines and myokines produced by muscular contractions [29,38].

Additionally, the contribution of gut microbiota alteration in the pathogenesis of IBD is generally accepted [39,40,41]. PA has been reported to be a significant modifier in preventing and restoring gut dysbiosis [42]; PA is associated with an increase in microflora diversity, an improvement in the development of commensal bacteria, and a beneficial metabolic function [42,43].

The psychological benefits of PA must also be considered [12]; PA may be useful in managing stress and anxiety, reducing depression, and developing self-esteem and character in IBD children [12,28].

Finally, benefits of PA on the bone mineral density (BMD) in children and adolescents with IBD should be also considered. As reported [44] have an increased risk to develop altered body composition, including low BMD. Regular PA may help to counteract the negative effects of disease on BMD [44]. The inclusion of PA in the therapeutic programs of IBD could be represent a strategical approach to maintain bone health and to reduce the risk of osteoporosis related to the combination of inflammation, malabsorption, and drugs such as corticosteroids. Personalized approaches, focused on individual choices and preferences, may be important especially for children who are not interested in PA and sports, regardless of the presence of IBD. Trainers could promote PA by tailoring to children’s needs the exercise session [45,46,47], and focusing the training on skills in which children feel most capable to increase enjoyment and, consequently, adherence to PA. For example, since we found that children with IBD have a higher perception of their speed agility and strength, trainers should enhance training programs focusing on anaerobic exercises, such as repeated sprint (with adequate recovery time) and strength exercises, to increase enjoyment and adherence to PA. Then, after regular practice of these exercises, trainers should reduce anaerobic and increase aerobic activities, to gradually reach an appropriate balance of training components. Unfortunately for trainers, one relevant problem in the promotion of PA in patients with IBD is the absence of guidelines and indications for IBD patients for PA practice in this population. However, considering that healthy style acquisition could track to later ages, a proposal of exercise programs for a healthy lifestyle starting in childhood could offer a better preventive strategy to preserve the health of children with IBD.

The limitations of the study include a limited number of participants; an increased sample size is expected to extend and validate these preliminary results on statistical and clinical grounds in the future. In this study, we only analyzed the severity of the disease as IBD-specific factors related to limited PA; other variables involved in impeding PA could be detailed, such as physical symptoms (pain and fatigue), complications, psychological factors (self-efficacy, self-perception, depression, and anxiety), and social support. Finally, no data on the physical activities prior to getting sick were recorded, and the low PA levels independently to disease could not be excluded. Further research is also needed to characterize how these factors impact on PA. The identification of key risk factors for low PA levels could inform the development of interventions to enhance PA in children and adolescents with IBD.

## 6. Conclusions

A poorer PA level and poor physical competence, including general and cardiorespiratory fitness, strength, speed agility, and flexibility, were found in patients affected by IBD compared to healthy children and adolescents. A correlation between PA levels and PGA and activity disease severity was also noted. An integrated treatment, including dedicated exercise programs, may be useful in providing tailored therapeutic strategies and preventing cardiometabolic comorbidities and other preventable disorders.

## Figures and Tables

**Table 1:** Clinical features, physical activity level, and fitness performance of patients with inflammatory bowel disease (IBD) and controls.

	IBD Patientsn = 54	Controlsn = 70	*p*	Adjusted p*
Age (years) mean (sd)	14.6 ± 2.2	13.5 ± 2.2	0.68	
Sex (M) N(%)	22 (40.7)	22 (32.3)	0.34	
Weight (kg) mean (sd)	55.9 (15.4)	45.9 (10.1)	<0.001	na
Height (cm) mean (sd)	150 (13)	156 (11)	0.12	na
BMI (kg/m2) mean (sd)	21.6 (4.3)	18.7 (3.4)	<0.001	na
BMI z-score median (IQR)	0.29 (−0.91–1.04)	−0.75 (−1.3–0.06)	0.002	na
Physical activity level mean (sd)	1.50 (0.34)	2.06 (0.42)	<0.001	<0.001
General fitness mean (sd)	3.05 (1.00)	3.55 (0.88)	0.003	0.13
Cardiorespiratory fitness mean (sd)	2.87 (1.10)	3.46 (1.00)	0.002	0.026
Strength mean (sd)	3.14 (1.05)	3.55 (0.85)	0.01	0.043
Speed agility mean (sd)	3.20 (1.20)	3.78 (0.99)	0.003	0.31
Flexibility mean (sd)	3.12 (1.01)	3.57 (1.03)	0.01	0.26

*p adjusted for BMI z-score, sex, and age; na = not applicable.

**Table 2:** Physical activity (PA) levels and physical competences in patients affected by IBD according to Physician Global Assessment (PGA) and Pediatric Ulcerative Colitis Activity Index (PUCAI)/Pediatric Crohn’s Disease Activity Index (PCDAI) were reported.

	PA Levels	General Fitness	Cardiorespiratory Fitness	Strength	Speed Agility	Flexibility
**PGA**						
1	1.57 (0.35)	2.96 (0.95)	3.0 (1.06)	3.15 (1.12)	3.24 (1.14)	3.12(0.89)
2	1.45 (0.30)	3.53 (1.05)	2.84 (1.21)	3.15 (1.14)	3.30 (1.49)	3 (1.29)
3	1.33 (0.24)	2.62 (0.91)	2.37 (1.06)	3.12 (0.64)	2.87 (0.99)	3.37 (1.06)
Total	1.50 (0.33)	3.05 (0.99)	2.87 (01.09)	3.14 (1.05)	3.20 (1.20)	3.12(1.01)
**PUCAI/PCDAI**						
inactive disease/remission	1.54 (0.33)	3.0 (0.98)	2.90 (1.16)	3.14 (1.13)	3.23 (1.20)	3.07 (0.99)
mild/moderate	1.37 (0.32)	3.25 (1.05)	2.75 (0.86)	3.16 (0.71)	3.08 (1.24)	3.33 (1.07)
Total	1.50 (0.33)	3.05 (0.99)	2.87 (1.09)	3.14 (1.05)	3.20 (1.20)	3.12 (1.01)

## Data Availability

The data presented in this study are available on request from the corresponding author. The data are not publicly available due to privacy reasons.
